# A puzzling renal Fanconi syndrome

**DOI:** 10.1093/ckj/sfae408

**Published:** 2025-01-10

**Authors:** Ludwig Haydock, Marguerite Hureaux, Maxime Hoffmann, Rosa Vargas-Poussou, Bertrand Knebelmann

**Affiliations:** Service de Néphrologie Adulte, Hôpital Necker-Enfants Malades, Assistance Publique, Hôpitaux de Paris (AP-HP) Université Paris Cité, Paris, France; Department of Medicine, Nephrology Research Group, Laval University, Quebec City, Quebec, Canada; Université Paris Cité, Inserm, PARCC, Paris, France; Service de Médecine Génomique, Hôpital Européen Georges Pompidou, Fédération de Génétique et de Médecine Génomique Assistance Publique-Hôpitaux de Paris Centre Université Paris Cité, Paris, France; Centre de Recherche Cardio-vasculaire de Paris, UMR970; Hôpital privé La Louvière, Groupe Ramsay Santé, Lille, France; Service de Médecine Génomique, Hôpital Européen Georges Pompidou, Fédération de Génétique et de Médecine Génomique Assistance Publique-Hôpitaux de Paris Centre Université Paris Cité, Paris, France; Service de Néphrologie Adulte, Hôpital Necker-Enfants Malades, Assistance Publique, Hôpitaux de Paris (AP-HP) Université Paris Cité, Paris, France

**Keywords:** EHHADH, Fanconi syndrome, GATM, HNF4A, tubulopathy

## Abstract

Renal Fanconi syndrome (FS) can be either acquired or inherited. When FS presents at a young age, it is typically inherited, with cystinosis being the most common cause. In this report we describe a rare cause of autosomal dominant Fanconi syndrome, Fanconi renotubular syndrome type 3 (FRTS3), caused by the already reported heterozygous p.E3K variant in the *EHHADH* gene. Only two FRTS3 families have been reported in the literature, and the kidney function was stated as normal or only slightly decreased into late life. Our family expands the spectrum of FRTS3, with some individuals showing only glucosuria and mild low-molecular-weight proteinuria, while others exhibited complete Fanconi syndrome with rickets. Importantly, we observed impairment of kidney function at a young age in our proband, highlighting a broader phenotypic variability associated with FRTS3.

## INTRODUCTION

Inherited forms of renal Fanconi syndrome (FS) typically present at a young age and may not always have a clear family history. This can be due to various factors, such as autosomal recessive inheritance with isolated cases, *de novo* mutations, X-linked transmission, mosaicism, or mild phenotypes in relatives that go unrecognized. We describe a young patient whose FS remained of unknown origin for many years. A definitive diagnosis was finally made after the discovery of a previously unrecognized positive family history, which motivated performing more extensive genetic analysis.

## CASE REPORT

**Figure 1: (a):**
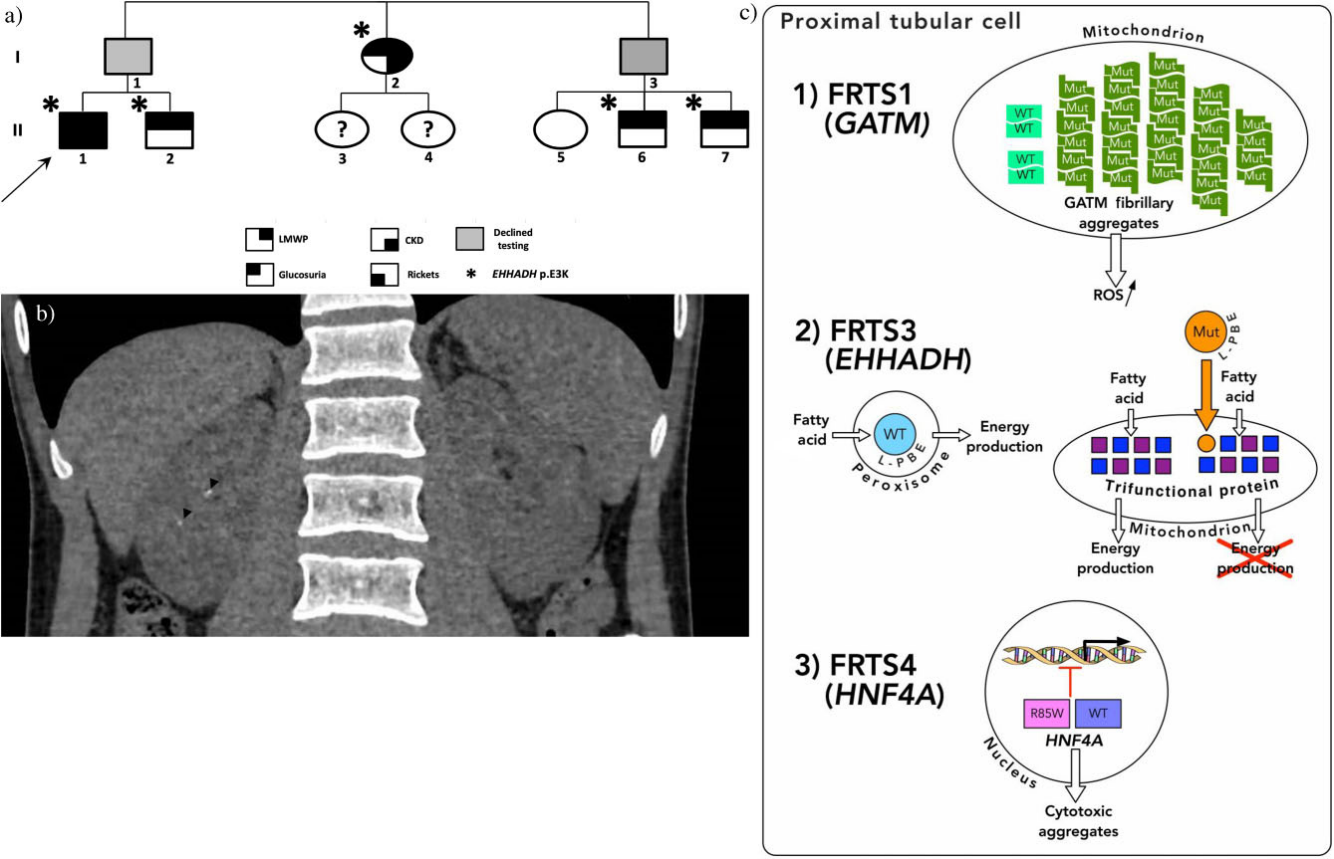
Family pedigree. Phenotypes of I-1 and I-3 were unknown because they declined investigations; however, they necessarily carry the *EHHADH* p.E3K variant. (**b**) Abdominal pelvic CT scan of the proband at the age of 25 revealed only millimetric nephrolithiasis (arrowheads) that have been stable for 5 years. (**c**) Genes implicated in autosomal dominant Fanconi syndromes. (1) Contrary to wild-type homodimers, mutant GATM forms multimers that aggregate in the mitochondria, increasing ROS production. (2) L-PBE protein encoded by *EHHADH* is normally directed to the peroxisome. By contrast, the *EHHADH* p.E3K variant encodes a mutant L-PBE protein that is misdirected to the mitochondrion, impairing normal β-oxidation of fatty acids and disrupting normal energy production. (3) The *HNF4A* p.R85W variant, in the DNA-binding domain, is believed to modify the transcription of genes regulated by *HNF4A*, and might promote the nuclear export of *HNF4A*, generating cytotoxic aggregates in the cytosol [3, 5]. WT, wild-type; Mut, mutant. DNA image under CC BY license.

A 20-year-old man (II-1) (Fig. 1a) was referred for proximal tubulopathy. His medical record started at 18 months when he was evaluated for failure to thrive, which led to the diagnosis of rickets. Laboratory tests showed complete proximal tubulopathy: hyperchloremic acidosis, hypophosphatemia, hypouricemia, glucosuria, low Tm of phosphate, hypercalciuria, aminoaciduria, and low-molecular-weight proteinuria (LMWP). Supplementation with bicarbonate, phosphate, and vitamin D improved rickets and allowed catch-up growth. Initial investigations were negative for cystinosis (normal intra-leukocyte cystine), tyrosinemia (normal blood tyrosine), Wilson disease (normal plasma ceruloplasmin), and Dent and Lowe disease (no *CLCN5* or *OCRL* causal variant). The FS remained of unknown origin for many years.

At the age of 20, when he transitioned to our adult nephrology department, his family history, which had previously not been relevant for any kidney disease, was further explored. It revealed a partial FS in his younger brother (II-2) and two paternal cousins (II-6, II-7) as well as in his paternal aunt (I-2), who also had impaired kidney function (eGFR 58 mL/min/1.73 m^2^ at 59 years) (Fig. 1a). The phenotypes of his father (I-1) and paternal uncle (I-3) were unknown because they declined investigations.

At the age of 23, eGFR was 74 mL/min/1.73 m^2^ and a kidney biopsy showed no specific glomerular lesion, few interstitial calcifications, and no anomaly of mitochondria (no aggregates, normal morphology) by electron microscopy.

Based on these new findings, a novel genetic analysis was conducted, focusing on recently discovered causal genes of FS. It revealed a likely pathogenic heterozygous *ENOYL-CoA HYDRATASE/3-HYDROXYACYL CoA DEHYDROGENASE* (*EHHADH*) (NM_001966.4) missense variant (c.7G > A; p.E3K). Segregation analysis subsequently revealed that all affected family members were also heterozygotes for this variant, enabling the diagnosis of FRTS3. Indeed, this same pathogenic variant has been reported in two FRTS3-affected families [1, 2],

At the last follow-up the proband was aged 31 years; under bicarbonates and vitamin D supplementation his eGFR was 73 mL/min/1.73 m^2^, bicarbonate level 23 mmol/L, ionized calcium 1.20 mmol/L, and phosphate 0.94 mmol/L. The nephrocalcinosis that was initially noticed at the age of 8 did not progress on subsequent imagery (Fig. 1b); however, bone mineral density decreased (lumbar spine *Z*-score −2.7DS ; femoral neck *Z*-score −1.8DS, and forearm *Z*-score −2.0DS), highlighting the dilemma between bone and kidney health in proximal tubulopathy.

## DISCUSSION

This family pedigree demonstrates an autosomal dominant transmission of FS, despite the proband initially denying any family history. This highlights the importance of not relying solely on the absence of clinical signs reported by patients about their relatives but also conducting thorough laboratory investigations and, when appropriate, DNA testing to end the diagnostic odyssey.

There are only three types of FS that have been described with an autosomal dominant mode of inheritance [3].

- FRTS1 caused by *L-ARGININE:GLYCINE AMIDINOTRANSFERASE* (*GATM*) missense variants that generate an abnormal interaction site on GATM protein which leads to the formation ofGATM multimers instead of the usual homodimer. These GATM multimers aggregate in the mitochondria and are seen on electron microscopy as fibril-like structures, giving the appearance of giant mitochondria, (Fig. 1c). Reported cases of FRTS1 were associated with renal failure occurring in the third to sixth decades of life [4].
- FRTS3 caused by the c.7G > A (p.E3K) variant in the *EHHADH* (NM_001966.4) gene, which encodes L-bifunctional enzyme (L-PBE), a peroxisomal enzyme involved in the β-oxidation of fatty acids. The pathogenic effect of the p.E3K variant is believed to be through the creation a new N-terminal domain that generates a mitochondrial targeting signal, instead of the normal C-terminal peroxisome targeting sequence. This impairs the normal β-oxidation of fatty acids by the mitochondrial trifunctional protein [1] (Fig. 1c).
- FRTS4 caused by the c.253C > T (p.R85W) variant in the *HNF4α* gene (NM_000457.4) (Fig. 1c) is generally accompanied by extrarenal features such as macrosomia, neonatal hypoglycemia, and/or diabetes [3, 5].

The peculiarity of autosomal dominant FS lies in its association with a few recurrent specific missense variants in the *GATM, EHHADH*, or *HNF4α* gene, which exhibit a dominant-negative effect on proximal tubular cell function. This entity is rare but kidney function in previously reported FRTS3 individuals was noted to be either normal or slightly decreased into late life [1, 2]. The impaired kidney function observed in both the proband, supported by a kidney biopsy that did not indicate any other specific cause, and his aunt, suggests that renal function could be affected in this disease. However, additional cases are required before establishing a definitive link between the *EHHADH* p.E3K variant and loss of kidney function.

In conclusion, our family pedigree illustrates the broad spectrum of FRTS3 with significant intrafamilial variability. Some individuals present only isolated glucosuria and mild LMWP, which may be overlooked during initial assessment, while others exhibit complete proximal tubulopathy, rickets, and impaired kidney function.

## PATIENT CONSENT

The authors declare that they have obtained written consent from the patient discussed in the report.

## Data Availability

Detailed data presented here are available upon reasonable request to the corresponding author.
